# Landscape and rodent community composition are associated with risk of hemorrhagic fever with renal syndrome in two cities in China, 2006–2013

**DOI:** 10.1186/s12879-017-2827-5

**Published:** 2018-01-12

**Authors:** Hong Xiao, Xin Tong, Ru Huang, Lidong Gao, Shixiong Hu, Yapin Li, Hongwei Gao, Pai Zheng, Huisuo Yang, Zheng Y. X. Huang, Hua Tan, Huaiyu Tian

**Affiliations:** 1 College of Resources and Environmental Sciences, Hunan Normal University, Changsha, 410081 China; 2 Key Laboratory of Geospatial Big Data Mining and Application, Changsha, Hunan Province 410081 China; 3 Hunan Provincial Center for Disease Control and Prevention, Changsha, 410005 China; 4 Center for Disease Control and Prevention of Beijing Military Region, Beijing, 100042 China; 5 Institute of Disaster Medicine and Public Health, Affiliated Hospital of Logistics University of Chinese People’s Armed Police Force (PAP), Tianjin, China; 6 Department of Occupational and Environmental Health, Peking University School of Public Health, Beijing, 100191 China; 7 College of Life Sciences, Nanjing Normal University, Nanjing, China; 8 School of Biomedical Informatics, the University of Texas Health Science Center at Houston, Houston, Texas USA; 9 State Key Laboratory of Remote Sensing Science, College of Global Change and Earth System Science, Beijing Normal University, Beijing, 100875 China

**Keywords:** Hantavirus infection, Hemorrhagic fever with renal syndrome, Landscape, Rodent community composition

## Abstract

**Background:**

Hemorrhagic fever with renal syndrome (HFRS) is a rodent-borne disease caused by hantaviruses. Landscape can influence the risk of hantavirus infection for humans, mainly through its effect on rodent community composition and distribution. It is important to understand how landscapes influence population dynamics for different rodent species and the subsequent effect on HFRS risk.

**Methods:**

To determine how rodent community composition influenced human hantavirus infection, we monitored rodent communities in the prefecture-level cities of Loudi and Shaoyang, China, from 2006 to 2013. Land use data were extracted from satellite images and rodent community diversity was analyzed in 45 trapping sites, in different environments. Potential contact matrices, determining how rodent community composition influence HFRS infection among different land use types, were estimated based on rodent community composition and environment type for geo-located HFRS cases.

**Results:**

*Apodemus agrarius* and *Rattus norvegicus* were the predominant species in Loudi and Shaoyang, respectively. The major risk of HFRS infection was concentrated in areas with cultivated land and was associated with *A. agrarius*, *R. norvegicus*, and *Rattus flavipectus*. In urban areas in Shaoyang, *Mus musculus* was related to risk of hantavirus infection.

**Conclusions:**

Landscape features and rodent community dynamics may affect the risk of human hantavirus infection. Results of this study may be useful for the development of HFRS prevention initiatives that are customized for regions with different geographical environments.

## Background

Hemorrhagic fever with renal syndrome (HFRS) is a rodent-borne disease caused by hantaviruses. Each hantavirus tends to be specific to a different rodent or insectivore host [1, 2]. Two dominant hantaviruses, Seoul virus (SEOV) and Hantaan virus (HTNV), carried by *Rattus norvegicus* and *Apodemus agrarius*, respectively, cause HFRS in China [3]. China is one of the countries most affected by hantaviruses (mainly HTNV and SEOV). The reported cases in China account for 90% of the total global burden of the HFRS [4–6]. HFRS has become an important public health problem in Asia. The mortality rates have reached 12% in some outbreaks [7]. In recent years, the incidence of HFRS has significantly decreased. However, 30,000–60,000 cases are reported annually in China [8]. Hunan Province is one of the most seriously affected areas in mainland China [2, 6, 9]. Since HFRS was first detected in Hunan Province in 1963, more than 90% of the cities in the province have reported cases [4, 10, 11]. Hunan Province has reported several hantavirus strains, predominantly SEOV, and various species of rodent host, including *A. agrarius*, *R. norvegicus*, *Mus musculus*, and *Rattus flavipectus* [12, 13]. All of these species can carry hantaviruses [14] and were found to carry and transmit hantavirus frequently in recent years [15].

Rodent population densities, virus prevalence in rodents, diversity of rodents, rodent community composition, and species distributions have important influences on HFRS transmission [16–22]. Different rodent species thrive in different habitats. For example, *A. agrarius* prefers humid and food-rich environments, and is found predominantly in forested regions and fields. *R. norvegicus* is abundant in residential areas, and is the main vector for zoonotic diseases in rural and urban populations [4]. The main routes of transmission to humans are inhalation of aerosolized urine or feces, contact with the saliva of infected rodents, or via contaminated food, all of which require humans and rodent hosts to share the same space [4, 23]. Previous studies revealed that land use influences HFRS transmission through the effect on the reservoir, host, and environmental conditions [6, 24]. To date, few studies have examined the relationships among landscape, rodent community composition and HFRS occurrence. In 2006–2008, the rodent density in different habitats and the prevalence of major rodent-borne diseases (including HFRS) in Nanchang City in Jiangxi Province were investigated and the risks of the rodent-borne diseases were assessed [25]; The spatial as well as temporal variation in the occurrence of HFRS is linked to geographic differences in the population dynamics of the reservoir rodents in different biomes of Europe [26]. These studies showed that studying the relationships among landscape, rodent community composition and HFRS occurrence are beneficial works to promote the progress of the understanding of HFRS epidemiology.

The first case in Shaoyang, one of the prefecture-level cities most seriously affected by HFRS in Hunan Province, was reported in 1965 [27]. In 2006, 135 cases were reported in Shaoyang, accounting for 24.1% of the total cases in Hunan Province. There were more than 1000 cases, in total, from 1980 to 1999, but the incidence decreased for unknown reasons by 54.3% during this time period. In prefecture-level city of Loudi, after the first case emerged in the 1970s, the incidence of HFRS increased substantially in the 1990s. Despite a decline in incidence in Loudi that began in the early 2000s, there was still one area with high incidence. The annual incidence in Loudi increased to 3.7 cases per 100,000 people in 2007, and was the highest in Hunan Province.

The aims of this study were to: 1) investigate how the community composition of the hosts influences the risk of HFRS among different landscapes; 2) to identify dominant rodent species in different environments; and 3) to investigate the spatiotemporal distribution of hantavirus infection risk at small spatial scales.

## Methods

### Study area

**Fig. 1:**
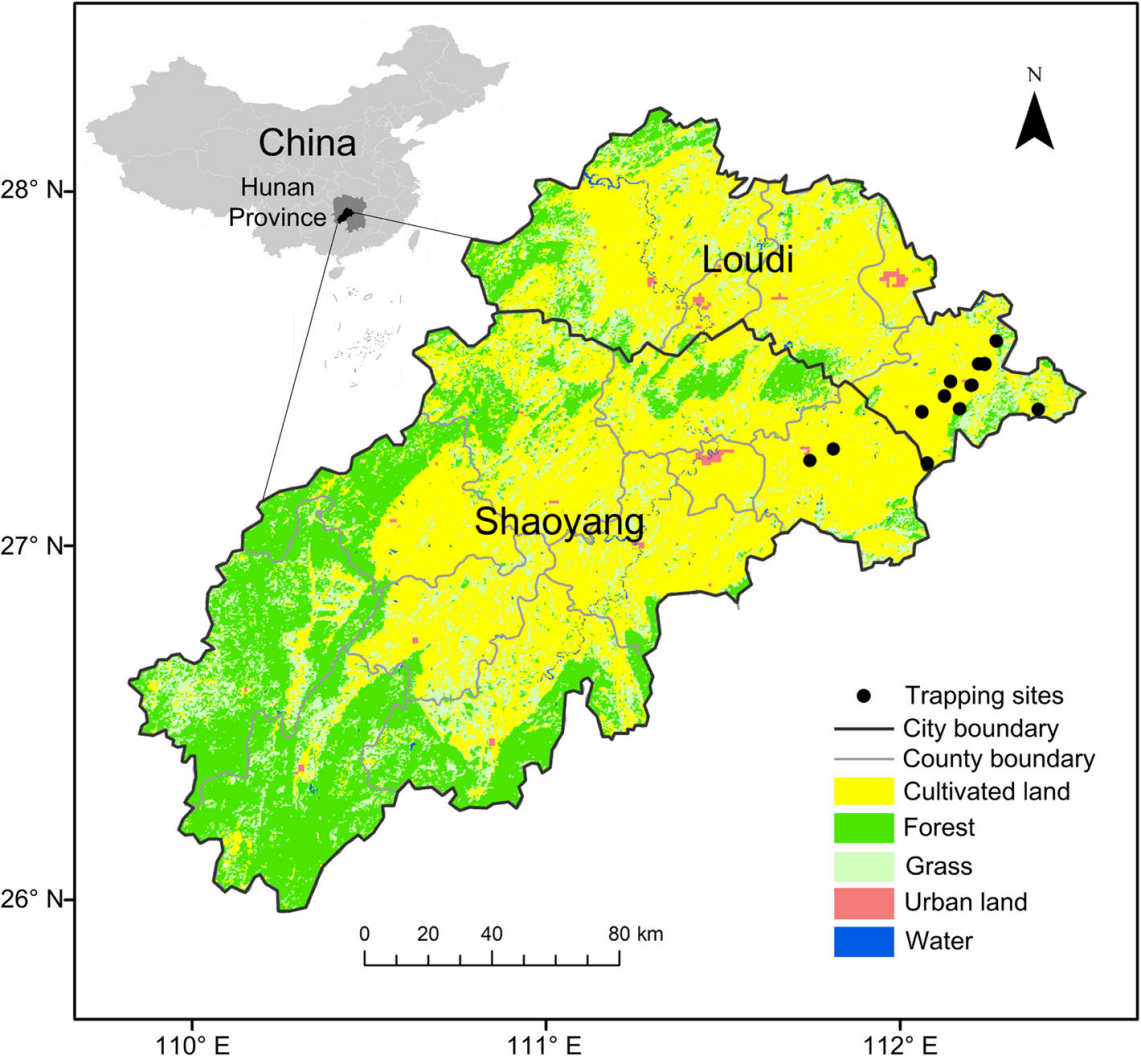
Land use and location of trapping sites in the study area, the prefecture-level cities of Loudi and Shaoyang

The study was conducted in the prefecture-level cities of Shaoyang and Loudi, in the southwest of Hunan Province (Fig. 1). Shaoyang has mainly mountainous terrain, an annual average temperature of 16.1–17.1 °C, and annual rainfall of 1000–1300 mm. Shaoyang has a total land area of 20,829 km^2^ and a population of about 7.1 million people. Loudi covers 8117 km^2^ and has a population of 4.67 million people. In Loudi, the mean annual temperature is about 16.5–17.5 °C, and the annual rainfall is about 1300–1400 mm.

### Data collection

Data on HFRS cases in Shaoyang and Loudi from 2006 to 2013 were obtained from the Hunan Notifiable Disease Surveillance System (HNDSS). The HNDSS is a passive surveillance system. All HFRS cases were first diagnosed based on clinical symptoms, as defined by a national standard [28]. The diagnosis was confirmed by detection of specific IgM and IgG antibodies to hantaviruses in acute phase serum specimens by enzyme-linked immunosorbent assay (ELISA). Information recorded for each case included sex, age, residential address, and the date of onset of symptoms. The HFRS data in this analysis did not differentiate HTNV from SEOV infections. Cases were geo-coded according to the residential address using Google Earth (Google, Mountain View, California, USA). As most patients’ occupations are farmer, their working places were mainly farmland, closing to their family address. We hypothesize that people usually have the most frequent activities near their address and working places. Thus the residential address of HFRS cases could reflect the environmental condition where the infected persons exposure to rodents.

The rodent monitoring data in Loudi and Shaoyang were collected by 45 permanent trapping sites covering different environments; 36 in cultivated areas and three, each, in forests, grasslands, and urban areas. As the trapping sites were geocoded at township-level, some sites in the same township are represented by one point in the map (Fig. 1). A total of 48,328 trap-nights occurred between 2006 and 2013. According to the HFRS monitoring program of Hunan, rodents were trapped in March, April, September, and October [29]. The traps were baited with peanuts, placed at each trapping site each night, and checked in the morning. More than 100 traps were placed per site in peridomestic environments, at approximately 12–15 m intervals, for 3 consecutive nights. More than 200 traps were placed per site outdoors, for 3 consecutive nights (every 5 m in each row, with 50 m between rows). The trapped rodents were numbered and the species and sex were identified.

Land use data were extracted from the GlobCover 2009 land cover map, provided by Université Catholique de Louvain (UCL) and ESA (http://due.esrin.esa.int/page_globcover.php [30]), with a resolution of 300 m. The original GlobCover 2009 global land cover data were collected by the Medium Resolution Imaging Spectrometer (MERIS) sensor data from the Envisat satellite. The study areas were categorized into five land use types, cultivated land, forest, grassland, urban land, and water bodies (such as rivers and lakes). Maps were created using ArcGIS 10.0 (ESRI Inc., Redlands, CA, USA).

### Statistical analysis

The same data analysis was conducted for Loudi and Shaoyang. First, the annual proportions of HFRS cases for the five land use types were calculated. A matrix (*R*) was constructed, with rows representing the proportion of HFRS cases for one land use type in different years, and columns representing the proportion of HFRS cases in the same year for different land use types. Second, the annual proportions of different rodent species were calculated based on rodent surveillance data. The rodents were classified mainly as *R. norvegicus*, *M. musculus*, *A. agrarius*, *R. flavipectus*, and other rodent species (including *Rattus losea* and *Microtus fortis calamorum*). The rodent community composition was quantified as matrix *C*, with rows representing the proportion of the same rodent species in different years, and columns representing the proportion of different rodent species in the same year. Elements of each column in matrix R and matrix C should add up to one. After that, the coefficient matrix *β* was calculated from *R* and *C* according to Eq. (1) using the method of matrix right division to determine how rodent community composition influences the HFRS occurrence probability:

1 $$ \left(\begin{array}{cccc}{R}_{11}& {R}_{12}& \cdots & {R}_{1j}\\ {}{R}_{21}& {R}_{22}& & \\ {}\vdots & & \ddots & \vdots \\ {}{R}_{i1}& & \cdots & {R}_{ij}\end{array}\right)=\left(\begin{array}{cccc}{\beta}_{11}& {\beta}_{12}& \cdots & {\beta}_{1k}\\ {}{\beta}_{21}& {\beta}_{22}& & \\ {}\vdots & & \ddots & \vdots \\ {}{\beta}_{i1}& & \cdots & {\beta}_{ik}\end{array}\right)\cdot \left(\begin{array}{cccc}{C}_{11}& {C}_{12}& \cdots & {C}_{1j}\\ {}{C}_{21}& {C}_{22}& & \\ {}\vdots & & \ddots & \vdots \\ {}{C}_{k1}& & \cdots & {C}_{\mathrm{kj}}\end{array}\right) $$

where *R*_*ij*_ is the proportion of HFRS cases in area *i* in year *j*, *β*_*ik*_ shows the potential contact rate of HFRS from rodent species *k* to humans in area *i*, and *C*_*kj*_ is the proportion of rodent species *k* in year *j*.

**Fig. 2:**
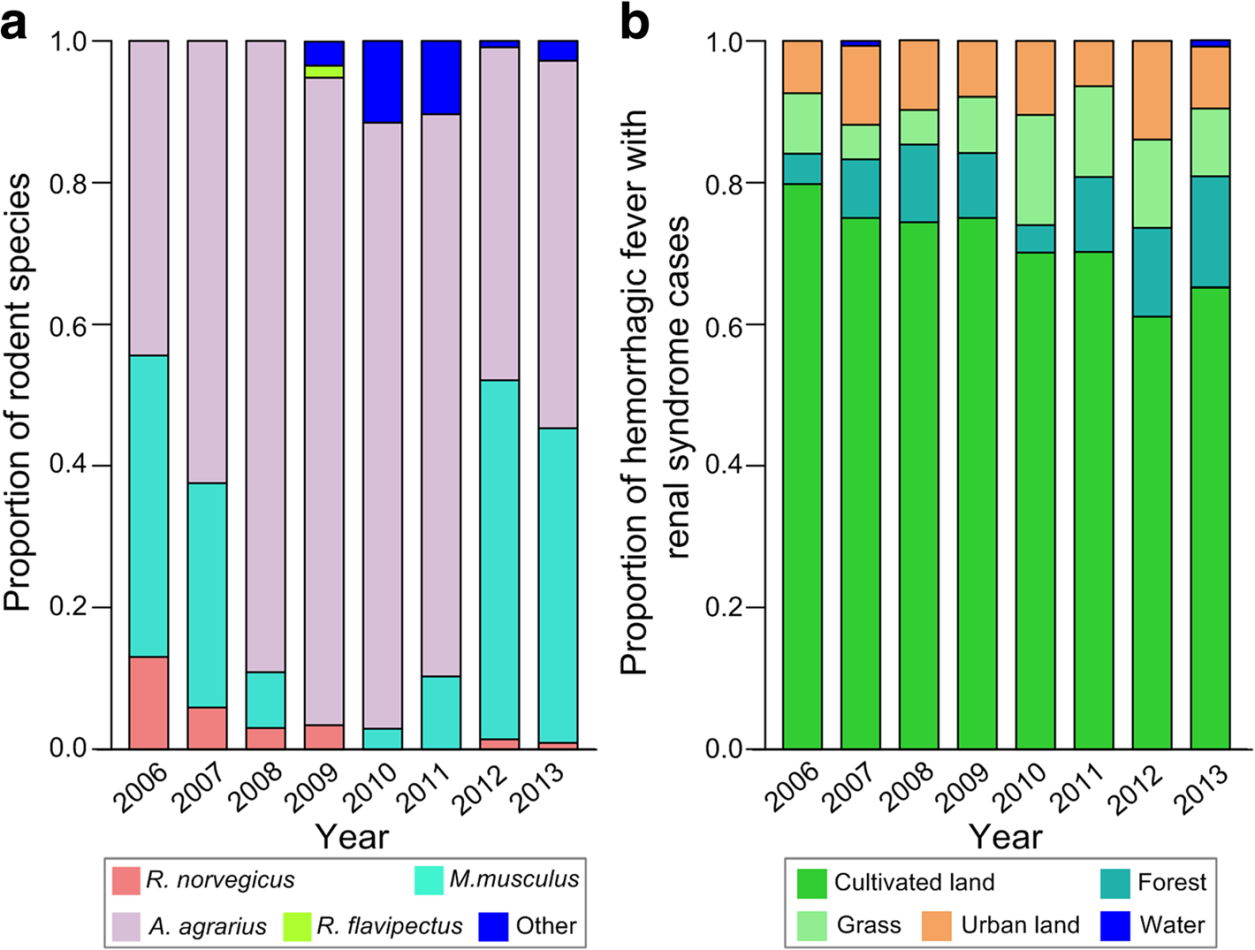
Visualized coefficient matrix showing the relationships among rodent community composition, land use types and HFRS occurrence in (**a**) Loudi, (**b**) Shaoyang. The coefficient values are color coded from blue (low values) to red (high values)

The *β* matrices for Loudi and Shaoyang are shown in Fig. 2, with low values in dark blue and high values in red. Each value in the figure is a coefficient for one rodent species and one land use type. All the values are dimensionless. Positive values represent positive association among the HFRS occurrence, rodent species, and land use types, and negative values represent negative associations.

All data were divided into two categories. Training data (75%), collected from 2006 to 2011, were used to develop the model and estimate the coefficient matrix. Validation data (25%), collected from 2012 to 2013, were used for model evaluation. The matrices *R* and *C*, constructed with data from 2006 to 2011, were used to calculate the coefficient matrix *β*. Based on the testing matrix *C*, constructed with data from 2012 to 2013, and the coefficient matrix *β*, we estimated the proportion of HFRS occurrence among different land use types in 2012–2013. The calculated results and the observed data from 2012 to 2013 in both Loudi and Shaoyang were used to perform a linear fitting to assess the accuracy of our predicted results. The accuracy of prediction was reflected by the R^2^ and was thought as better when the R^2^ was closer to 1. All statistical analyses were performed using SPSS 19 software (SPSS Inc., Chicago, IL, USA) and Matlab (vR2012b) (Math Works Inc., Natick, MA, USA).

## Results

### Species distribution and HFRS occurrence

**Fig. 3:**
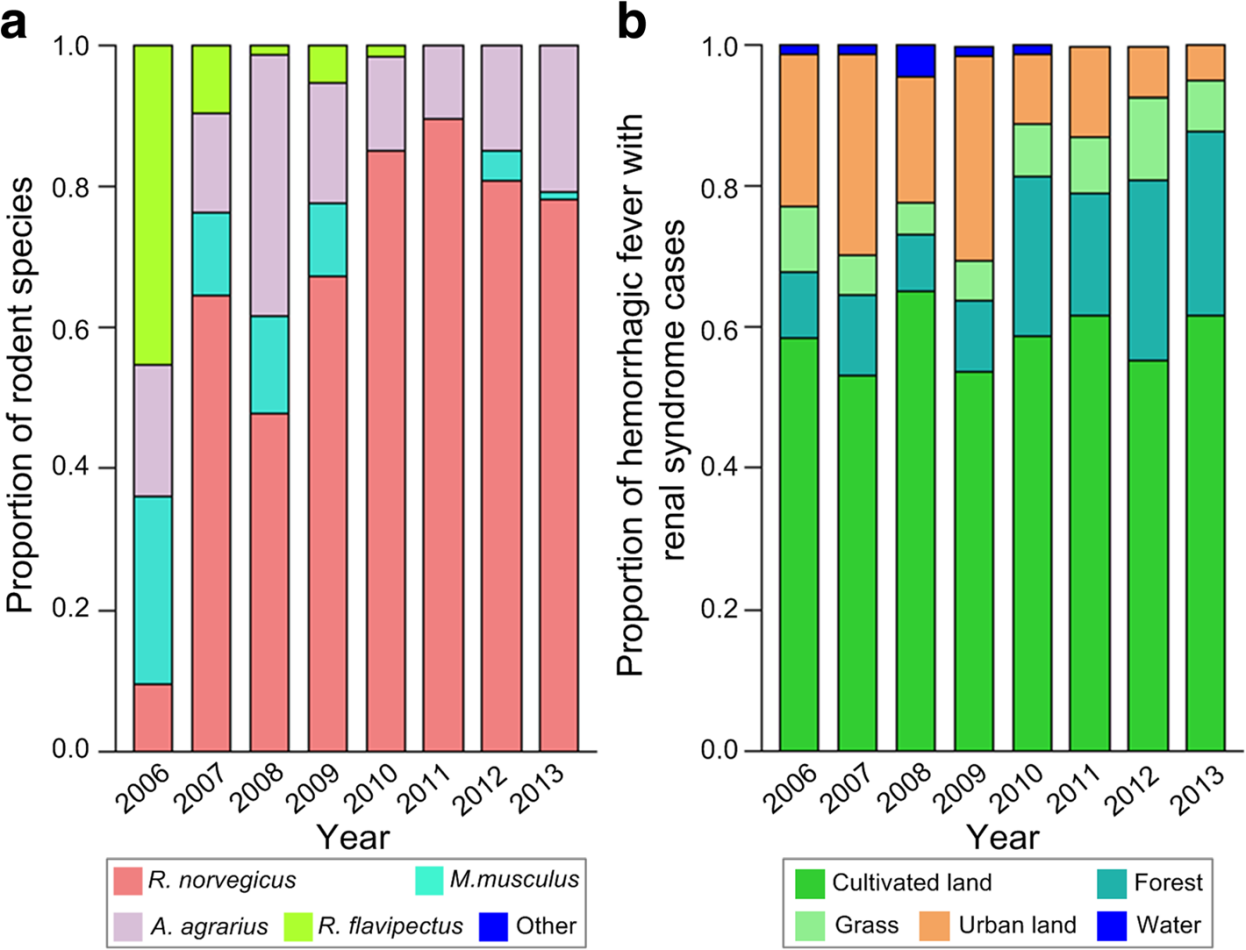
Distribution of rodent species and HFRS cases in Loudi, 2006–2013. **a** Proportion of each rodent species, (**b**) Proportion of HFRS cases among different land use types

**Fig. 4:**
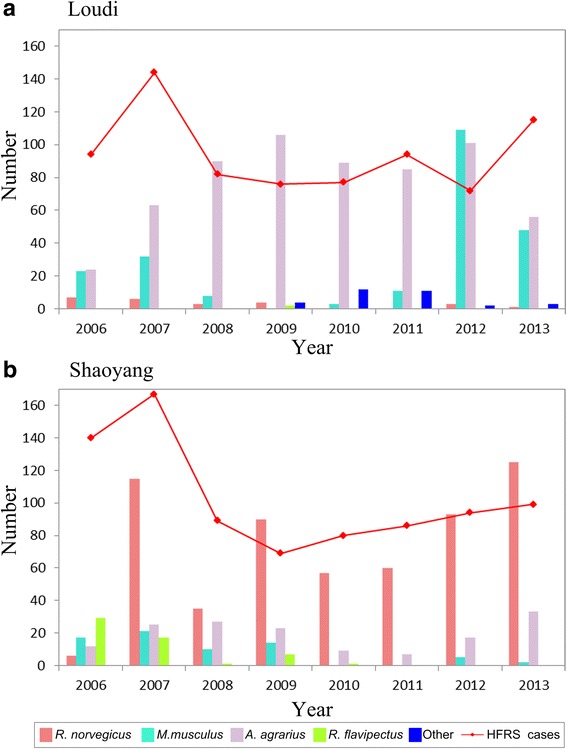
Number of rodents trapped and HFRS cases reported in (**a**) Loudi, (**b**) Shaoyang

A total of 906 rodents were trapped in Loudi, where *A. agrarius*, the main reservoir of HTNV, was the predominant species, accounting for 91.4% of all trapped rodents in 2009. The number of *M. musculus* from 2006 to 2013 varied, with none trapped in 2009 and 109 trapped in 2012. The number of *R. norvegicus* decreased yearly from 2006 and none were trapped in 2010 and 2011. Other species (mainly *Rattus losea and Microtus fortis calamorum*) were trapped starting in 2009 (Figs. 3a and 4a).

A total of 742 HFRS cases were confirmed in Loudi between 2006 and 2013. Figure 4a shows the annual distribution of cases. More cases occurred in 2007 than in any other year. The number of HFRS cases declined from 2007 to 2010. Case reports increased in 2011 and 2013, with 94 and 115 cases reported, respectively (Fig. 4a). There was little annual variation in the proportion of HFRS cases for each land use type in Loudi (Fig. 3b). Cultivated land consistently had the largest proportion of cases. The distributions of HFRS cases in urban areas, forests, and grasslands were similar. Except in 2007 and 2013, no cases occurred in bodies of water. In 2010, consistent with the increase in other rodent species, there was an increase in HFRS cases in grassland areas.

**Fig. 5:**
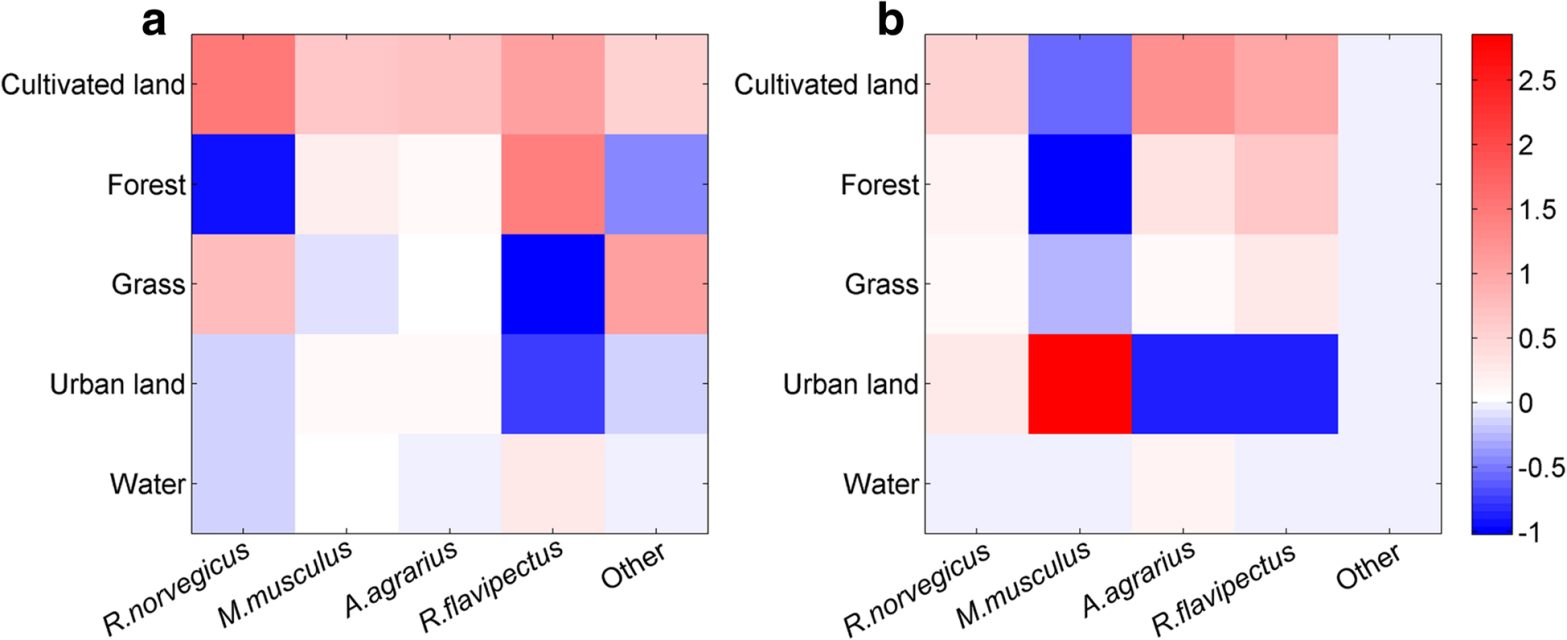
Distribution of rodent species and HFRS cases in Shaoyang, 2006–2013. **a** Proportion of each rodent species, (**b**) Proportion of HFRS cases among different land use types

In Shaoyang, a total of 858 rodents were trapped during the study period. *R. norvegicus* (67.7%) and *A. agrarius* (17.8%) were the predominant rodent species. *R. flavipectus* and *M. musculus* accounted for 71.9% of all trapped species in 2006, but this proportion declined over the next 7 years. In 2010 and 2011, no *M. musculus* were trapped, but they appeared again in 2012 and 2013. No *R. flavipectus* were trapped from 2011 to 2013. There were no other rodent species trapped in Shaoyang (Figs. 5a and 4b).

Shaoyang had 797 HFRS cases during the study period and the incidence was highest in 2007. The number of cases declined in 2008 and increased between 2009 and 2013 (Fig. 4b). The proportion of cases for each land use type varied over the study period. Cultivated land consistently had the highest proportion of cases. In urban areas, the proportion of cases declined annually. However, annual occurrence increased in forests. There was little variation in HFRS cases in grassland areas over the study period. There were a few HFRS cases reported in bodies of water from 2006 to 2010, but no cases were reported in these areas after 2010 (Fig. 5b).

### Relationships between rodent hosts, land use types, and HFRS occurrences

The coefficient matrix, *β*, identified the potential influence of rodent species distributions in different land use types on the occurrence of HFRS. In Loudi, HFRS cases in cultivated land were positively associated with all rodent species. In forests, HFRS cases were positively associated with *R. flavipectus* and *M. musculus* and negatively associated with *R. norvegicus* and other rodent species. In grasslands, HFRS cases were positively associated with *R. norvegicus* and other rodent species and negatively associated with *R. flavipectus* and *M. musculus*, while the opposite occurred in forests. In urban land and water bodies, HFRS cases were negatively associated with *R. norvegicus* and other rodent species. There was a weak positive association of *M. musculus* and *A. agrarius* with HFRS cases in urban land, and *R. flavipectus* was negatively associated with HFRS cases. In water bodies, there was a weak negative association of HFRS cases with *A. agrarius* and a positive association with *R. flavipectus* (Fig. 2a). In Shaoyang, HFRS cases were positively associated with *R. norvegicus*, *A. agrarius*, and *R. flavipectus*, and negatively associated with *M. musculus* in cultivated land, forest, and grassland. In urban land, HFRS cases were positively associated with *R. norvegicus* and *M. musculus*, and negatively associated with *A. agrarius* and *R. flavipectus*. In water bodies, there was a weak negative association of all rodent species, except *A. agrarius*, with HFRS cases. Additionally, there was a weak association of other species with HFRS cases in all land use types (Fig. 2b).

### Risk of potential contacts between humans and rodents in different land use types

**Fig. 6:**
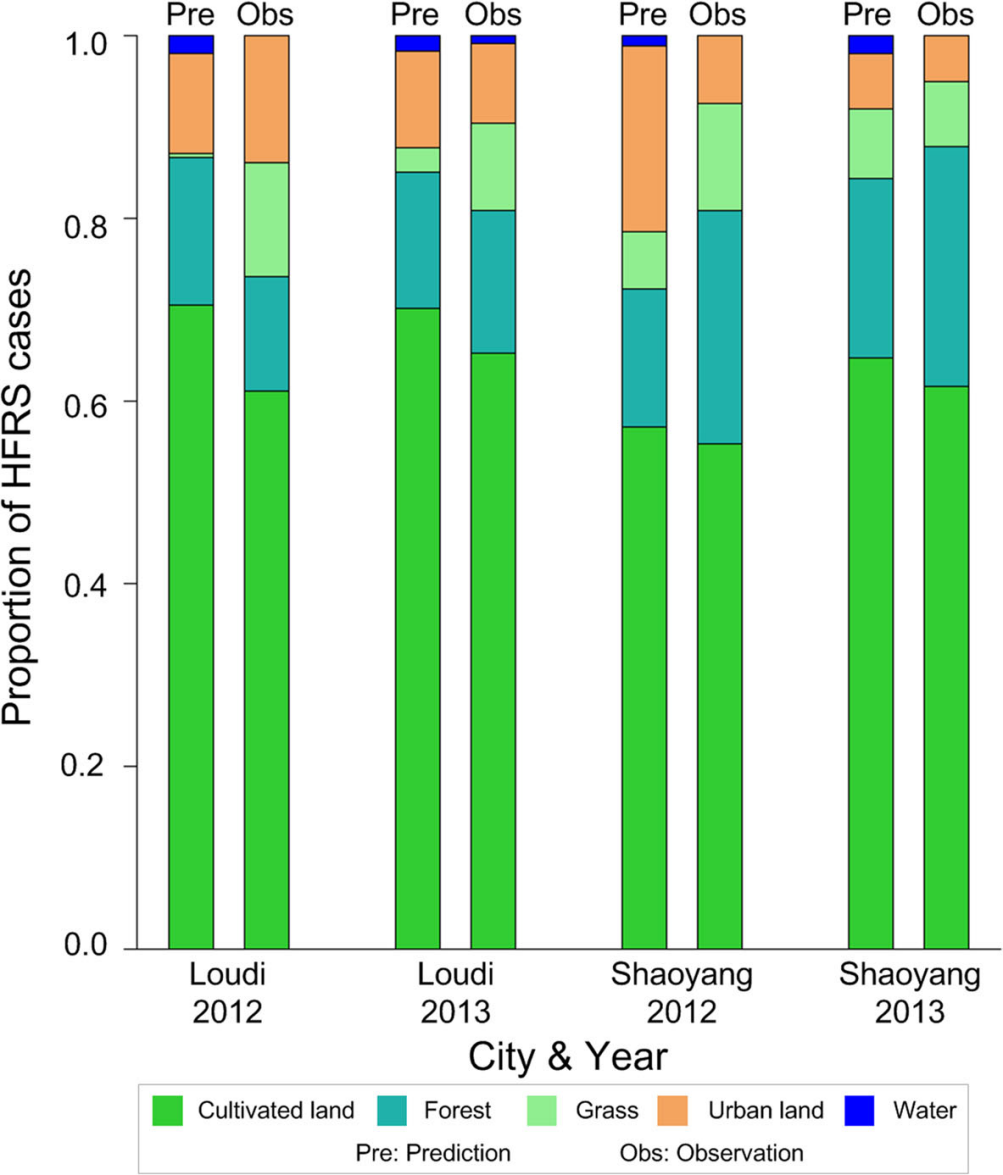
Predicted and observed HFRS occurrence probability among different land use types in Loudi and Shaoyang, 2012–2013. The HFRS occurrence probability is predicted by Eq. 1

**Fig. 7:**
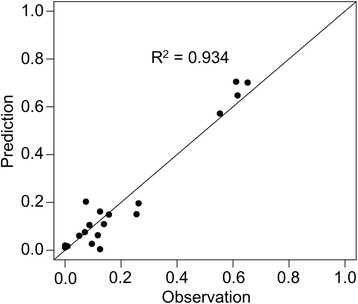
Scatterplot showing the predicted and observed HFRS occurrence probabilities. The HFRS occurrence probability is predicted by Eq. 1

The proportions of HFRS cases among different land use types in Loudi and Shaoyang in 2012–2013 were predicted. In Loudi, the land use type with the highest predicted risk of HFRS was cultivated land, following by forest and urban land in both 2012 and 2013. The model predicted that grassland and water bodies would have a low risk of HFRS in these 2 years. In Shaoyang, cultivated land had the highest predicted risk of HFRS in both years, followed by urban land, forest, grassland, and water bodies in 2012, and followed by forest, grassland, urban land, and water bodies in 2013. Figure 6 shows the predicted probability of occurrence of HFRS cases as well as the corresponding observations in both Loudi and Shaoyang in 2012–2013. The predicted and observed proportions in the same area in the same year were paired to assess the accuracy of the predictive model. The scatterplot in Fig. 7 shows the concordant relationship of the predictions and observations. The R^2^ reflected that our prediction was accurate (R^2^ = 0.934).

## Discussion

This study investigated the relationships among HFRS occurrence, land use type, and rodent community composition. The results indicated that different rodent species influenced the HFRS occurrence for different land use types.

Overall, the highest probability of HFRS was on cultivated land, followed by urban areas, forests, and grasslands. Relatively few cases of HFRS were identified in water covered areas in both Shaoyang and Loudi. For the same land use type, the probability of HFRS occurrence varied between cities. The high probability of HFRS on cultivated land may be due to the humid environment with adequate food for rodents to survive. Farmers working on cultivated land increase the potential for contact between rodents and humans, thereby increasing the risk of HFRS transmission. In 2012, relatively high HFRS risk was predicted in urban areas in Shaoyang, but in 2013, the predicted HFRS risk was lower. This might have resulted from increases in *R. norvegicus* and *A. agrarius* in 2012 and 2013. *R. norvegicus* was positively associated with HFRS in urban land while *A. agrarius* was negatively associated with the HFRS in urban land. The negative correlation was stronger than the positive correlation (Fig. 2b). Therefore, the increase in *A. agrarius* had a greater influence on HFRS in urban land in Shaoyang. An increase in intensive human activities, such as farming, leading to agricultural encroachment on forests, grassland areas, and water covered areas, and large human populations in urban areas changing the geographical landscape [20], has an important impact on the spread of viruses. A previous study, focused on HFRS cases caused by HTNV in rural areas, found that agricultural land use and cultivated soil were related to high risk for HFRS [6]. We found the risks among different land use types varied in relation to rodent community composition.

Hantaviruses are transmitted to humans by infected rodents. Different land uses lead to different rodent community composition and distribution [31, 32]. Moreover, each land use type has a predominant rodent species. In the current study, the risks of hantavirus infection in cultivated land were associated with different rodent species in Loudi and Shaoyang. The risks of HFRS occurrence in other land use types varied for different rodent species. In Loudi, *A. agrarius* was the most predominant species (Figs. 3 and 4a). However, the highest risk of hantavirus infection was on cultivated land, and mainly correlated with *R. norvegicus* (Fig. 2a)*.* This suggests Loudi city may be a mixed-type epidemic area. In Shaoyang, *R. norvegicus* was the predominant species (Figs. 5 and 4b). Cultivated and urban areas had higher risk of HFRS and HFRS in these areas was predominantly associated with *A. agrarius* and *M. musculus*, respectively (Fig. 2b), indicating that Shaoyang may be a mixed-type epidemic area. It can be concluded that both of the cities are mixed-type HFRS epidemic areas with various reservoir rodents. The corresponding risks of potential contact between humans and rodents in different landscapes may also change over time with varied rodent community composition.

Different rodent population dynamics have disparate influences on HFRS occurrence. *A. agrarius* and *R. norvegicus* were the predominant species in Loudi and Shaoyang, respectively. According to monitoring data from the last 20 years in China, the highest virus-carrying indexes among host animals in wild and residential areas are for *A. agrarius* and *R. norvegicus*, respectively [33–35]. Different rodents have their own preferred habitats and different abilities to carry and transmit pathogenic viruses. *A. agrarius* are more active outdoors and *M. musculus*, *R. norvegicus*, and *R. flavipectus* are active both outdoors and indoors [13]. We found different rodent species in both Loudi and Shaoyang, so the occurrences of HFRS cases in both outdoor (cultivated land, forest, grass, and water) and indoor (urban land) environments are consistent with prior knowledge. The coefficient matrix of Loudi indicated that *R. norvegicus* was the dominant species affecting HFRS risk on cultivated land. HFRS occurrence and the related dominant rodent host varied for each environment. This suggests that rodent community composition has a significant influence on the epidemic pattern and transmissions of hantaviruses.

Based on these findings, preventative measures can be developed for different land use types, in different cities and epidemic areas. HFRS is related to the number of rodents in different environments. Therefore, we can effectively identify the dominant rodent species in different areas and enact preventative measures to reduce the risk of hantavirus transmission. Cultivated land was a high-risk area for HFRS in our study. The dominant rodent species in this environment has an important impact on the HFRS risk. Therefore, more attention should be spent reducing rodent numbers in these environments. This is consistent with a previous study that found that HFRS cases commonly occur in rural areas [36]. *R. norvegicus* was the main vector for hantavirus in Loudi and the main vectors in Shaoyang were primarily *A. agrarius*, *R. flavipectus*, and *M. musculus*. Differences in rodent community composition may result in different epidemic characteristics, infection risks, and even control measures. For example, when *A. agrarius* is the predominant species in the rodent population, as in Loudi, the main risk of HFRS is from cultivated land, so the prevention of HFRS should focus more on the farmlands. In contrast, when *R. norvegius* is the predominant species in the rodent population, such as in Shaoyang, the main risk of HFRS is from cultivated land, forest, and urban land, which indicates that more attention should be paid to all these types of land. Our study indicates that rodent community composition and land use types are associated with the epidemiology of HFRS. This information can be used to develop species-specific control measures to reduce the risk of potential contact between hantavirus and humans in different environments.

Several limitations for this study should be noted. First, it only considered the influence of rodents on HFRS. Hantavirus transmission results from a combination of environment, climate change, change in biotope, hantavirus species, and social factors [13, 31, 37]. Second, more detailed information about both rodents and humans needs to be considered, including rodent density, virus-carrying index, and population density. In the absence of the virus-carrying and population density data, we cannot investigate the actual role of rodent species in viral transmission from rodents to humans. Third, change in land use was not considered in our model because these data were not available. Finally, we used the postal addresses of patients to represent the sites of contact, which might have induced measurement error. The accuracy of address resolution was also limited. Further studies are needed to determine the effect of rodent community composition, density, distribution and virus-carrying index on the risks of HFRS transmission. Additionally, potential seasonal variation in prevalence is critical and should be considered when studying contact rate. It is therefore prevalence, seasonal variations of prevalence, and other environmental factors should also be considered in future studies.

## Conclusions

This study identified the dominant rodent species for different land use types in areas with HFRS, and provides support for the development of regional rodent monitoring programs to prevent HFRS in different environments. We also found that change in rodent community composition was associated with risk of hantavirus infection in different land use types. In addition, this study provides baseline data for HFRS incidence in Loudi and Shaoyang, China.

## Abbreviations

HFRS: Hemorrhagic fever with renal syndrome
HNDSS: Hunan notifiable disease surveillance system
HTNV: Hantaan virus
SEOV: Seoul virus
